# Successful laparoscopic treatment for sustained abdominal pain due to fish bone migrating into the neck of the pancreas: a case report and thinking about surgical approach through the literature review

**DOI:** 10.1186/s40792-021-01174-y

**Published:** 2021-04-13

**Authors:** Yang Wang, Xianzhang Luo, Jiefeng Zhang

**Affiliations:** Hepatic Biliary and Pancreatic Cancer Center, Chongqing University Cancer Hospital, No. 181 Hanyu Road, Shapingba, Chongqing, 400030 People’s Republic of China

**Keywords:** Fish bone, Pancreatic neck, Laparoscopic surgery, Case report

## Abstract

**Background:**

The majority of ingested foreign bodies pass through the gastrointestinal tract smoothly, with less than 1% requiring surgery. Fish bone could perforate through the wall of stomach or duodenum and then migrate to other surrounding organs, like the pancreas and liver.

**Case presentation:**

We report herein the case of a 67-year-old male who presented with sustained mild epigastric pain. Abdominal computed tomography revealed a linear, hyperdense, foreign body along the stomach wall and pancreatic neck. We made a final diagnosis of localized inflammation caused by a fish bone penetrating the posterior wall of the gastric antrum and migrating into the neck of the pancreas. Upper gastrointestinal endoscopy was performed firstly, but no foreign body was found. Hence, a laparoscopic surgery was performed. The foreign body was removed safely in one piece and was identified as a 3.2-cm-long fish bone. The patient was discharged from the hospital on the fifth day after surgery without any postoperative complications.

**Conclusion:**

Laparoscopic surgery has proven to be a safe and effective way to remove an ingested fish bone embedded in the pancreas.

## Background

The ingestion of foreign bodies occurs commonly in clinical practice. The majority of ingested foreign bodies pass through the gastrointestinal tract smoothly, with approximately 10–20% of foreign bodies requiring an endoscopic procedure, and less than 1% requiring surgery [1]. Fish bone could perforate through the wall of stomach or duodenum and then migrate to other surrounding organs, like the pancreas and liver [2–6]. The penetration of fish bones into the pancreas is quite rare [3, 4]. Rapid diagnosis and prompt treatment are mandatory to improve the prognosis of this rare condition. A mortality rate of 10% has been reported because of missed or delayed diagnosis [7]. Thus, we herein report a case of laparoscopic removal of an ingested fish bone migration to the neck of pancreas.

## Case presentation

**Fig. 1:**
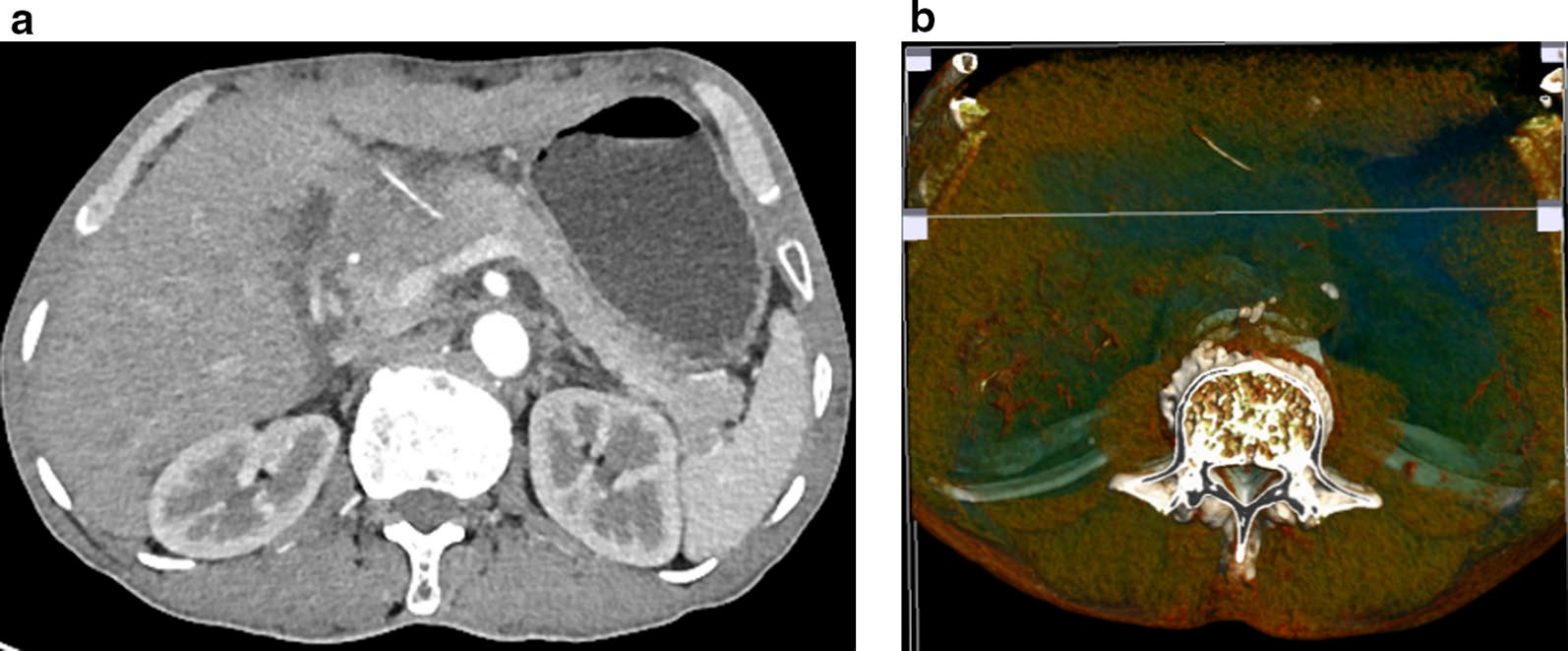
**a** Computed tomography scan of the abdomen revealed a linear, hyperdense, foreign body along the stomach wall and pancreatic neck. **b** The bone condition CT clearly shows the position and shape of the fish bone in the abdominal cavity

**Fig. 2:**
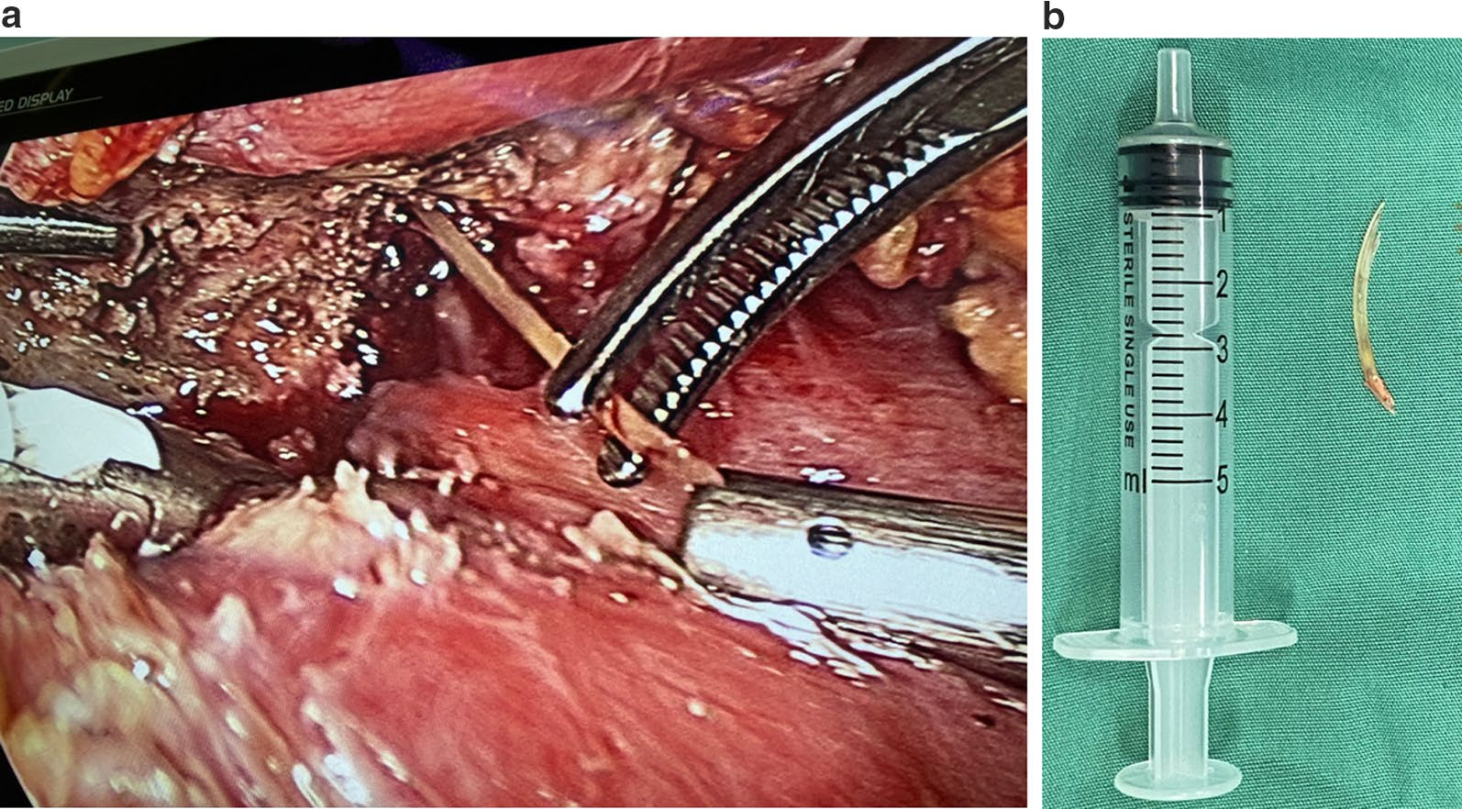
**a** A linear foreign body was found between the prepyloric region of the stomach and the pancreatic neck and was safely removed from both pancreas and stomach laparoscopically. **b** The foreign body was identified as a 3.2-cm-long fish bone after removal

A 67-year-old male patient was admitted to the gastroenterology department due to abdominal pain over 3 months. He was hospitalized with a diagnosis of gastricism and a proton-pump inhibitor was started, but abdominal pain persisted. Physical examination showed mild epigastric tenderness. A complete blood count on admission were as follows: white blood count 9.76 × 10^9^/L, the C-reactive protein level 138.31 mg/L, red blood count 3.79 × 10^12^/L, hemoglobin 120 g/L, platelets 118 × 10^9^/L, liver function tests, kidney function tests and pancreatic enzyme levels were within normal limits. Abdominal computed tomography (CT) was scheduled revealing a linear, hyperdense, foreign body along the stomach wall and pancreatic neck (Fig. 1a), and bone condition CT clearly shows the position and shape of the fish bone in the abdominal cavity (Fig. 1b). The patient was questioned about her past medical history. He remembered that he had abdominal pain after eating fish and something else 3 months ago. After urgent consultation, we made a final diagnosis of localized inflammation caused by a fish bone penetrating the posterior wall of the gastric antrum and migrating into the neck of the pancreas. Upper gastrointestinal endoscopy was performed; however, in addition to the chronic atrophic gastritis and distal gastric ulcer, no foreign body was found. Later, the patient was transferred to department of hepatobiliary and pancreatic oncology and underwent laparoscopic surgery. The patient was placed in a supine position. The operator stood on the right side of the patient, the assistant on the left side, and the scopist between the patient’s legs. Five trocars were placed: one above the navel for the laparoscopy (10 mm), two in the upper right abdominal quadrant (12 mm, 5 mm), and one in the upper left abdominal quadrant (10 mm, 5 mm). Fibrous structures were observed between the small curvature of the stomach and pancreas neck, and after the adhesions were dissected, a fish bone was identified and removed laparoscopically (Fig. 2a). The foreign body was identified as a 3.2-cm-long fish bone (Fig. 2b). Bleeding was controlled by pressure with a hemostatic gauze, and no suture repair was performed, because the penetrated wall was small and no leak was observed in both stomach and pancreas. Surgical intervention was completed after placing a drain in the operation area. The operation time is 2 h, and the bleeding during the operation is about 100 ml. Postoperative antibiotherapy was started, with proton-pump inhibitor treatment continuing for three more days. Clear fluid was drained, finally the drain pipe was removed on the third day after surgery. The patient was discharged from the hospital on the fifth day after surgery without any postoperative complications. And CT reexamination had not found obvious abnormality 2 months after the surgery.

## Discussion

Sharp foreign bodies, like fish bones, chicken bones, sewing needles and tooth picks, may be ingested spontaneously [8, 9]. Having been reported in less than 1% of the cases, gastrointestinal perforation may cause peritonitis, localized abscess or inflammatory mass, bleeding or fistula [5, 10–13]. Fish bone is one of the most commonly ingested foreign bodies [14]. In most cases, a fish bone penetrated the stomach or the duodenum, but rarely migrated into the pancreas [3, 7, 15, 16]. This injury may be presented as a suppurative infection or pancreatic mass of the pancreas [12, 13].

Rapid diagnosis and early intervention of gastrointestinal foreign bodies are required to prevent morbidity and mortality [3, 4, 17]. Generally, patients are unable to provide a clear history of fish bone ingestion. Useful for detecting an ingested fish bone and its associated complications, CT usually reveals a linear, hyperdense, foreign body corresponding to a bone [18]. Since numerous foreign bodies migrate to the pancreas, surgical removal was quite effective in the management of an ingested foreign body when an endoscopic removal failed [3, 4, 6]. In addition, a laparoscopic approach may be more beneficial than open procedures because it allows the surgeon to approach the lesser sac with minimal manipulation of surrounding tissues under the help of optimal magnification and illumination [3, 19]. Recent years have witnessed more and more similar cases being addressed through laparoscopic surgery [3, 4, 6]. We refer to the English literature and found that only nine cases of an ingested fish bone that penetrated through the digestive tract and was embedded in the pancreas [3, 4, 6, 7, 12, 13, 15, 16, 20], as demonstrated in Table 1. Patients underwent laparoscopic surgery were found to recover faster. Compared with cases underwent open surgery, their postoperative day discharge was significantly shorter, as shown in Table 2. Therefore, laparoscopic approach should be preferred especially in this series, due to its advantages of less postoperative pain, lower incidence of wound infection, and minimal surgical stress [21].

**Table 1:** Cases of an ingested fish bone that penetrated through the gastrointestinal tract and was embedded in the pancreas

Reference number	Author	Year	Location	Duration of the onset to diagnosis (day)	Surgery	Fish bone length (cm)	POD discharge (day)
13	Goh BK	2004	Stomach	14	Open	2.8	11
12	Wang WL	2008	Stomach	28	Open	2.3	8
20	Yasuda T	2010	Duodenum	3	Open	4	14
16	Symeonidis D	2012	Duodenum	2	Open	3	7
7	Huang YH	2013	Stomach	1	Open	3.2	12
15	Gharib SD	2015	Duodenum	18	Open	3.7	11
3	Kosuke Mima	2018	Stomach	1	Laparoscopic	2.5	7
6	Rui Xie	2019	Stomach	5	Laparoscopic	3.5	7
4	Francesk Mulita	2020	Stomach	2	Laparoscopic	3	4
–	Yang Wang	2020	Stomach	90	Laparoscopic	3.2	5

*POD* postoperative day

**Table 2:** Baseline characteristics and surgical treatment outcomes of patients

Characteristics	Total	Laparoscopic	Open
	(*n* = 10)	(*n* = 4)	(*n* = 6)
Year range of case report	2004–2020	2018–2020	2004–2015
Duration of the onset to diagnosis (days)			
Mean ± SD	16.4 ± 27.4	24.5 ± 43.7	11 ± 10.88
Location			
Stomach	7	4	3
Duodenum	3	0	3
Fish bone length (cm)			
Mean ± SD	3.12 ± 0.522	3.05 ± 0.42	3.167 ± 0.615
POD discharge (days)			
Mean ± SD	8.6 ± 3.238	5.75 ± 1.5	10.50 ± 2.588

*POD* postoperative day

## Conclusion

Our patient, after undergoing a laparoscopic removal of an ingested fish bone, recovered without complications. In short, laparoscopic surgery has proven to be a safe and effective way to remove an ingested fish bone embedded in the pancreas.

## Data Availability

Data and material are available in this case report.

## Abbreviations

CT: Computed tomography
